# LcSHMT4 from Sheepgrass Improves Tolerance to Cadmium and Manganese and Enhances Cd and Mn Accumulation in Grains

**DOI:** 10.3390/plants15010091

**Published:** 2025-12-27

**Authors:** Jianli Wang, Guili Di, Yuanyuan Lin, Linlin Mu, Xu Zhuang, Dongmei Zhang, Weibo Han, Tuanyao Chai, Aimin Zhou, Kun Qiao

**Affiliations:** 1 Institute of Forage and Grassland Sciences, Heilongjiang Academy of Agricultural Sciences, Harbin 150086, China; jianli@haas.cn (J.W.); mulinys@163.com (L.M.); 13030086918@163.com (X.Z.); zhd_mei@163.com (D.Z.); alclever@163.com (W.H.); 2 Industrial Crops Institute, Heilongjiang Academy of Agricultural Sciences, Harbin 150086, China; diguili59@163.com; 3 College of Horticulture and Landscape Architecture, Northeast Agricultural University, Harbin 150030, China; linyuanyuan2020@126.com; 4 College of Life Science, University of the Chinese Academy of Sciences, Beijing 100049, China; tychai@ucas.ac.cn

**Keywords:** sheepgrass, serine hydroxymethyltransferase, heavy metal stress, tolerance, transport

## Abstract

Heavy metal contamination is a serious environmental problem worldwide, with substantial negative ecological and economic effects. Serine hydroxymethyltransferase (SHMT) is a key metabolic and photorespiratory enzyme in plant cells, and it is also involved in stress responses. In this study, *LcSHMT4* was isolated from sheepgrass (*Leymus chinensis* (Trin.) Tzvel) after transcriptome sequence analysis. The transcript levels of *LcSHMT4* in sheepgrass seedlings increased under Cd and Mn stresses, and subcellular localization analysis in tobacco leaves revealed that its encoded protein localizes at the mitochondria. Transgenic yeast and rice lines overexpressing *LcSHMT4* showed increased tolerance to Cd and Mn, compared with that of their controls. In addition, compared with the control, transgenic rice overexpressing *LcSHMT4* accumulated more Cd and Mn in brown rice grains. The transcript levels of genes encoding Cd or Mn transporters were increased in the *LcSHMT4*-overexpressing transgenic rice lines. We speculate that LcSHMT4 may enhance Cd and Mn tolerance by increasing the activities of antioxidant enzymes and the glutathione content and increase heavy metal accumulation by inducing the expression of genes encoding transporters. These results highlight useful genetic resources and provide a theoretical basis for further research on heavy metal tolerance and the phytoremediation of heavy-metal-contaminated soil.

## 1. Introduction

Heavy metal contamination in the environment mainly results from human activities such as urban industrial wastewater discharge, coupled with poor pollution control and insufficient funds for environmental restoration. Heavy metal pollution poses a serious threat to the stability of the ecological environment [1,2]. Crop plants that absorb heavy metals from the soil show decreases in their yield and quality. Moreover, heavy metals can accumulate in the human body through the food chain, and excessive levels of these metals can damage organs and increase the risks of various diseases, including heart and lung diseases, osteoporosis, multiple fractures damage, Alzheimer’s disease, damage to the liver, brain, bones, kidney, respiratory system and nervous system, and other tissues, hypertension, joint and muscle pain, memory loss, emotional disorders, reproductive problems, and others [3,4,5]. Because soils are often contaminated with multiple heavy metals that interact with each other, it is difficult to address this problem. Among the remediation methods for heavy-metal-polluted soils, growing plants that absorb and accumulate heavy metals has the advantages of being effective and reasonably inexpensive. Thus, the cultivation of plants that absorb or transport heavy metals from the soil and accumulate them in above-ground parts has become the most important method for remediating heavy-metal-contaminated soils [6,7].

In recent years, growing attention has been paid to heavy metal contamination and strategies to mitigate heavy metal phytotoxicity. Previous studies have revealed several mechanisms of heavy metal transport and detoxification in plants. There are four main defense mechanisms: (1) cell wall binding; (2) cytoplasmic detoxification; (3) vacuolar sequestration; and (4) the oxidative stress response, where reactive oxygen species (ROS) levels surge under heavy metal stress. The ROS generated in response to heavy metal stimuli play a dual role; initially, they function as signaling molecules but, when they accumulate to excess, they damage cellular components [8]. The major ROS production sites inside plant cells include mitochondria and chloroplasts, with superoxide (O_2_^−^) and hydrogen peroxide (H_2_O_2_) being the predominant forms [9]. To maintain ROS at non-toxic levels, plants use two scavenging systems: (1) enzymatic antioxidants—including catalase (CAT), ascorbate peroxidase (APX), and superoxide dismutase (SOD)—and (2) non-enzymatic antioxidants—such as glutathione (GSH), flavonoids, and ascorbic acid (AsA) [10].

Serine hydroxymethyltransferase (SHMT) was first identified in 1946 by Shemin [11]. Although SHMTs have been extensively studied in humans and animals—where they are linked to diseases like cancer and ischemic stroke—plant SHMTs are not as well characterized [12]. *Arabidopsis* contains seven *SHMT* genes encoding three isoforms of SHMT proteins: cytosolic (cSHMT), mitochondrial (mSHMT), and chloroplastic SHMTs [13]. The mSHMT plays an important role in photorespiration by enhancing the interconversion of serine and glycine, while generating N5,N10-methylene tetrahydrofolate to sustain the photorespiratory cycle [14].

Analyses of gene expression under abiotic stress revealed strong induction of *AtSHMT3* expression by heat stress and of *AtSHMT5* by cold stress, suggesting that these two genes encode *SHMTs* involved in thermotolerance in *Arabidopsis thaliana* [15]. The tobacco (*Nicotiana tabacum*) genome contains 16 *NtSHMT* genes that can be grouped phylogenetically into four subfamilies [16]. Promoter analyses have revealed cis-elements (ABRE, DRE, MYB, MYC, and G-box) responsive to abscisic acid, jasmonic acid, and various stress signals in the promoter regions of *SHMT* genes. Analyses of their expression under drought and cold stress indicated that *NtSHMT3*, 4, 7, 9, and 10 are drought-responsive, while *NtSHMT13–16* are cold-responsive. Notably, *NtSHMT10* was found to be upregulated by drought, salt, and cold stress, highlighting its potential role in stress adaptation [16]. Further functional studies in rice (*Oryza sativa*) demonstrated that OsSHMT4—localized to the nucleus—was highly expressed in roots under Cd stress [17]. Knockout of *OsSHMT4* enhanced Cd tolerance while increasing selenium accumulation in shoots and grains of brown rice, suggesting that it encodes an enzyme with a dual role in selenium enrichment and Cd detoxification. These findings underscore the diverse roles of SHMTs in plant stress responses, although their precise molecular mechanisms in heavy metal detoxification warrant further investigation.

Sheepgrass (*Leymus chinensis* (Trin.) Tzvel), also known as alkali grass, is a perennial herbaceous plant [18]. It is primarily distributed in arid and semi-arid grasslands and is the dominant grass species in the Songnen Plain of northeastern China and the eastern regions of Inner Mongolia. Sheepgrass is rich in nutrients, with a robust root system and large biomass, making it an excellent forage for livestock [19,20]. Previous studies have shown that sheepgrass can thrive in soils contaminated with heavy metals such as cadmium, lead, zinc, and copper, as well as in coal-mining areas [21]. In addition, another study on its ability to translocate heavy metals also showed that sheepgrass can effectively absorb cobalt, manganese, copper, nickel, and lead from the soil. Furthermore, when grown with bio-organic fertilizers, sheepgrass showed strong potential to remediate heavy-metal-contaminated saline-alkali soils [22].

Recent studies have explored the mechanisms of heavy metal tolerance in sheepgrass. At the molecular level, the phenylpropane biosynthesis pathway and the tricarboxylic acid (TCA) cycle interact to regulate the secretion of organic acids from the roots, a process that decreases heavy metal toxicity. In addition, genes encoding certain catalytic enzymes were found to be upregulated in the roots of sheepgrass under heavy metal stress [23]. One of the upregulated genes, *LcNRAMP2*, encodes a transporter protein that sequesters heavy metal ions into vacuoles, thereby reducing their toxic effects and increasing heavy metal (Mn and Cd) tolerance [24]. However, few genetic resources related to heavy metal tolerance are available, so more research on functional genes involved in heavy metal resistance is required.

In this study, *LcSHMT4* was isolated after analyzing transcriptome sequence data of sheepgrass seedlings under Cd stress. The functions of LcSHMT4 were preliminarily explored through stress tolerance analyses, determination of ion contents, and measurement of antioxidant enzyme activity. The results of this study provide new information about the detoxification mechanism of LcSHMT4 under heavy metal stress. Our results highlight genetic resources that can be used to generate plants that tolerate and accumulate heavy metals, with potential applications in the remediation of heavy-metal-contaminated soil.

## 2. Results

### 2.1. Analysis of SHMT4 Sequence

The full-length nucleotide sequence of *LcSHMT4* contained 1416 bp, encoding 471 amino acids (accession number: PV719638). The sequence alignment showed that the protein sequences of LcSHMT4 had higher similarity with SHMT4s from other species. The MEME predicted results contained five similar conserved motifs (Figure S1, red box). A phylogenetic tree was constructed using MEGA5.0 software, revealing that LcSHMT4 was closely related to HvSHMT4 (*Hordeum vulgare*), TaSHMT4 (*Triticum aestivum*), and TdSHMT4 (*Triticum dicoccoides*) (Figure S2).

### 2.2. Cd and Mn Tolerance of Yeast Strains Expressing LcSHMT4

**Figure 1:**
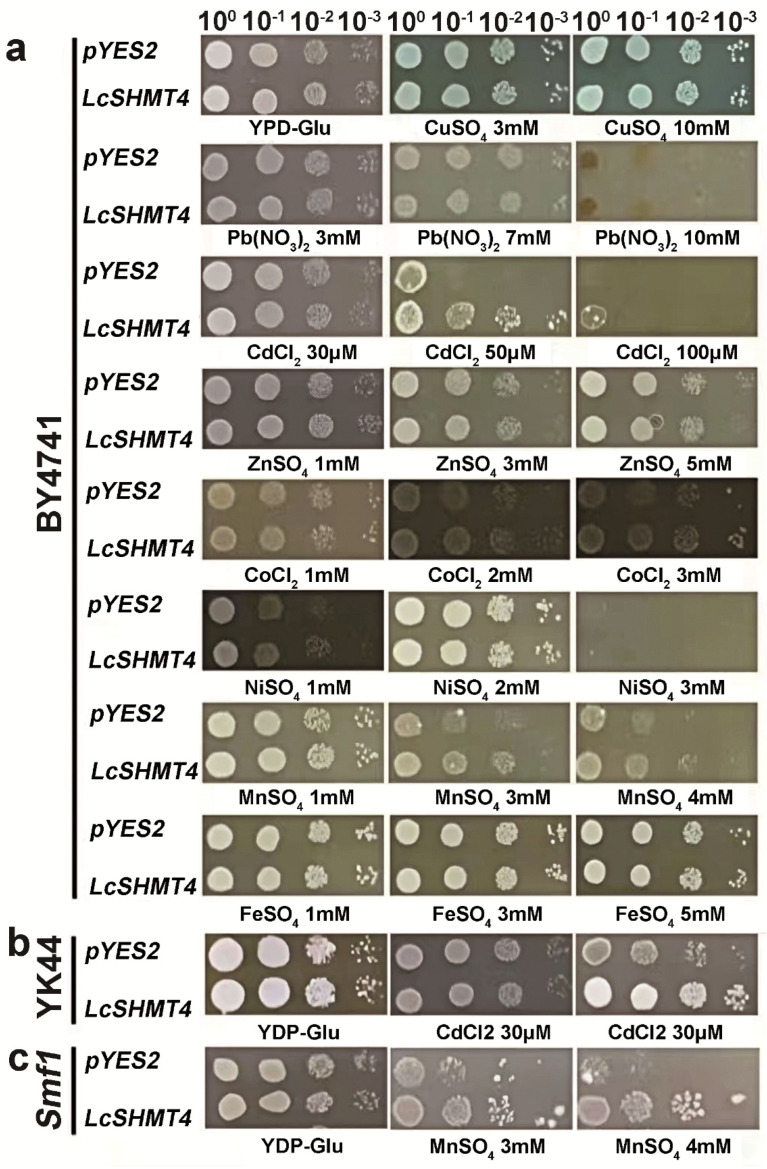
Heavy metal tolerance analysis of wild-type yeast (BY4741) and BY4741 overexpressing *LcSHMT4*, Cd-sensitive (YK44), and Mn-sensitive (*smfI*) yeast strains, and Cd–Mn-sensitive strains overexpressing *LcSHMT4*. (**a**) Tolerance of wild-type BY4741 and transgenic BY4741 overexpressing *LcSHMT4* was tested against eight heavy metals (3 and 10 mM CuSO_4_, 3, 7, and 10 mM Pb(NO_3_)_2_, 30, 50, and 100 μM CdCl_2_, 1, 3, and 5 mM ZnSO_4_, 1, 2, and 3 mM CoCl_2_, 1, 2, and 3 mM NiSO_4_, 1, 3, and 4 mM MnSO_4_, and 1, 3, and 5 mM FeSO_4_). Tolerance of YK44 (**b**) and *smfI* (**c**) strains and their transgenic lines expressing *LcSHMT4* was tested against Cd and Mn (30 and 50 μM CdCl_2_ and 3 and 4 mM MnSO_4_). The empty vector pYES2 (control) and pYES2-LcSHMT4 were transformed into BY4741, YK44, and *Smf1* using the PEG/LiAC method. All yeast cultures were adjusted to OD_600_ = 0.5 (10^0^), 10^−1^, 10^−2^, and 10^−3^. A total of 4 μL of each dilution was cultured on the yeast extract/peptone/galactose (YPG) solid media containing different concentrations of heavy metals. The growth of yeast cells was observed and photographed at 30 °C for 2–7 days.

Yeast cells transformed with the empty pYES2 (control) and *LcSHMT4* were spotted onto yeast extract/peptone/glucose (YPD) medium, and both strains exhibited similar growth after 36 h. The *LcSHMT4*-expressing transgenic yeast grew significantly better than the control under 50 and 100 μM CdCl_2_ and 3 and 4 mM MnSO_4_ but showed similar growth to that of the control in the Cu, Pb, Zn, Co, Ni, and Fe treatments (Figure 1). To further validate the ability of LcSHMT4 to enhance the Cd and Mn tolerance of yeast cells, we evaluated the growth of the yeast strains YK44 (Cd-sensitive) and *smf1* (Mn-sensitive) transformed with the empty vector (control) or LcSHMT4 on YPG medium supplemented with CdCl_2_ and MnSO_4_. The *LcSHMT4*-transformed yeast strains YK44 and *smf1* exhibited significantly better growth than the controls under 50 μM CdCl_2_ and 4 mM MnSO_4_ stress (Figure 1), demonstrating that LcSHMT4 improves the Cd and Mn tolerance of both wild-type and metal-sensitive yeast strains.

**Figure 2:**
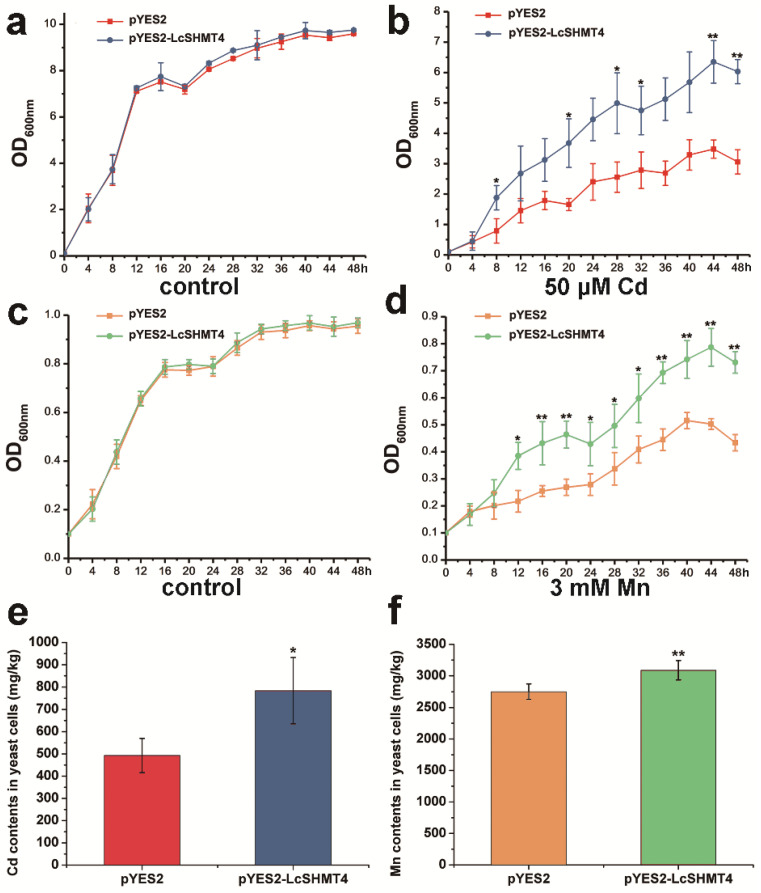
Growth curves and Cd or Mn accumulation of sensitive yeast strains overexpressing *LcSHMT4*. Initial yeast cell concentration was adjusted to 0.1 (OD_600_ = 0.1) and growth curves were determined by measuring change in OD_600_ from 0 to 48 h. YK44 yeast was cultured in YPG media containing 0 (**a**) and 50 μM CdCl_2_ (**c**). *smfI* yeast was cultured in YPG media containing 0 (**b**) and 3 mM MnSO_4_ (**d**). Cd (**e**) contents in YK44 and Mn (**f**) contents in *SmfI* transformed with empty vector (pYES2) or pYES2-LcSHMT4 under Cd or Mn treatments. Asterisks indicate significant differences (* *p* < 0.05, ** *p* < 0.01; *t*-test).

To explore the degree of heavy metal tolerance conferred by LcSHMT4 on yeast, we analyzed the growth curve of the control (transformed with pYES2) and transgenic lines. The growth curve of *LcSHMT4*-expressing YK44 cells was identical to that of the control in the absence of Cd or Mn (Figure 2a,b). However, in YPG supplemented with 50 μM CdCl_2_, both strains were able to grow, but the *LcSHMT4*-expressing YK44 cells grew significantly better than the control cells from 8 h to 48 h (Figure 2c, *p* < 0.05). Similarly, under 3 mM MnSO_4_ stress, the *LcSHMT4*-expressing *smfI* cells grew consistently better than the control cells from 12 h to 48 h, with statistically significant differences (Figure 2d, *p* < 0.05).

Under CdCl_2_ stress, the *LcSHMT4*-expressing YK44 cells accumulated approximately 1.8-fold more Cd compared with control cells (Figure 2e, *p* < 0.05). Under MnSO_4_ stress, the *LcSHMT4*-expressing *smfI* cells accumulated more Mn than pYES2 cells (Figure 2f, *p* < 0.01).

### 2.3. Expression of LcSHMT4 Under Cd and Mn Stresses

**Figure 3:**
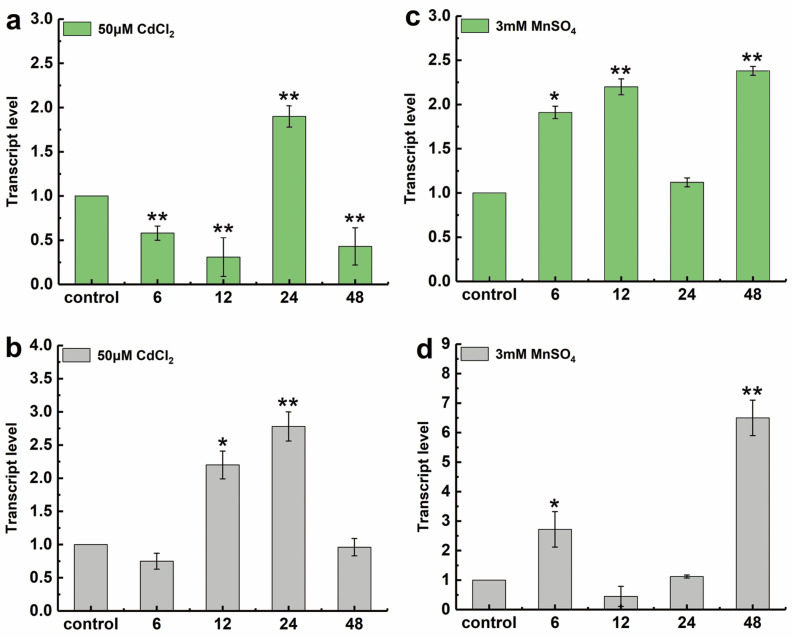
Transcript levels of *LcSHMT4* in sheepgrass seedlings subjected to 50 μM CdCl_2_ and 3 mM MnSO_4_ at 0, 6, 12, 24, and 48 h. (**a**,**b**) 50 μM CdCl_2_, (**c**,**d**) 3 mM MnSO_4_. (**a**,**c**) shoots, and (**b**,**d**) roots. Values are mean ± standard error from three independent replicates. Asterisks indicate significant differences (* 0.01 < *p* < 0.05, ** *p* < 0.01; *t*-test). Five-day-old sheepgrass seedlings (shoot and root) were treated and collected with 50 μM CdCl_2_ and 3 mM MnSO_4_ at 0 h, 6 h, 12 h, 24 h, and 48 h. Total RNA was extracted and cDNA was synthesized. The transcript level of *LcSHMT4* was determined by RT-qPCR. Relative gene transcript levels were calculated using the 2^−ΔΔCT^ method as previously described.

Sheepgrass seedlings were subjected to 48 h heavy metal treatments, and the relative transcript levels of *LcSHMT4* in the shoots and roots were determined by RT-qPCR. Under 50 μM CdCl_2_ stress, the transcript level of *LcSHMT4* in the shoots initially decreased at 6 and 12 h and then reached the peak at 24 h at a level significantly higher than that in the control (0 h) but distinctly decreased at 48 h (Figure 3a, *p* < 0.01). In the roots, *LcSHMT4* transcript levels increased at 12 and 24 h, reaching a level 2.8-fold that in the control (0 h) at 24 h, but reduced at 48 h (Figure 3b, *p* < 0.01).

Under 3 mM MnSO_4_ stress, the *LcSHMT4* transcript levels in the shoots showed an upward trend at 6 and 12 h (*p* < 0.05), decreasing at 24 h, peaking at 48 h at ~2.3-fold that in the control (Figure 3c, *p* < 0.01). In the roots, the transcript level sharply increased by 6 h to a level ~2.7-fold that in the control (*p* < 0.05), then decreased at 12 and 24 h, and increased again by 48 h to a level ~6.6-fold that in the control (Figure 3d, *p* < 0.01).

### 2.4. Localization of LcSHMT4 at the Mitochondria

**Figure 4:**
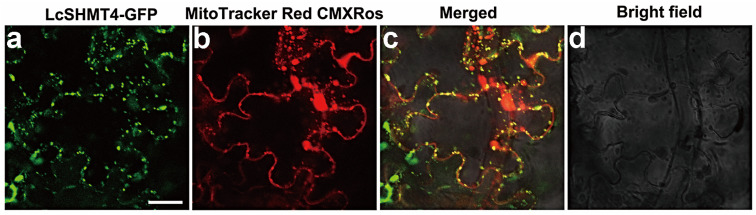
Subcellular localization of LcSHMT4 in tobacco leaves. Green signal, LcSHM4-eGFP; red signal, MitoTracker Red CMXRos; yellow signal, merged green and red signals. Scale bars: 20 μm.

To elucidate the functional mechanism of the LcSHMT4 protein in plants, its subcellular localization was determined in a transient expression assay. Tobacco leaves transiently expressing *LcSHMT4* were stained with the mitochondrial red dye MitoTracker Red CMXRos, and the expression and localization of the pCAMBIA1300-LcSHMT4-eGFP fusion protein were observed under a confocal laser scanning microscope. The dye and fusion protein signals largely overlapped in the tobacco cells, indicating that the LcSHMT4 protein localized at the mitochondria. This suggests that LcSHMT4 may have similar functions and mechanisms of action as other SHMT proteins localized at the mitochondria (Figure 4) [25].

### 2.5. Cd and Mn Tolerance of Rice Overexpressing LcSHMT4

The pCAMBIA1300-LcSHMT4-eGFP recombinant plasmid was sent to Weimi Biotechnology Co., Ltd. (Taizhou, China), who generated multiple transgenic rice lines using genetic transformation techniques. Semi-qPCR and RT-qPCR were used to measure the transcript levels of the transgene in six transgenic lines. *LcSHMT4* was amplified from all six transgenic lines (OE1–6) (Figure S3a), and the *LcSHMT4* transcript levels were confirmed to be higher in the OE1–6 lines than in WT. The OE1, OE2, and OE6 lines exhibited higher *SHMT4* transcript levels at 150-fold, 160-fold, and 60-fold that in WT, respectively (Figure S3b, *p* < 0.001).

**Figure 5:**
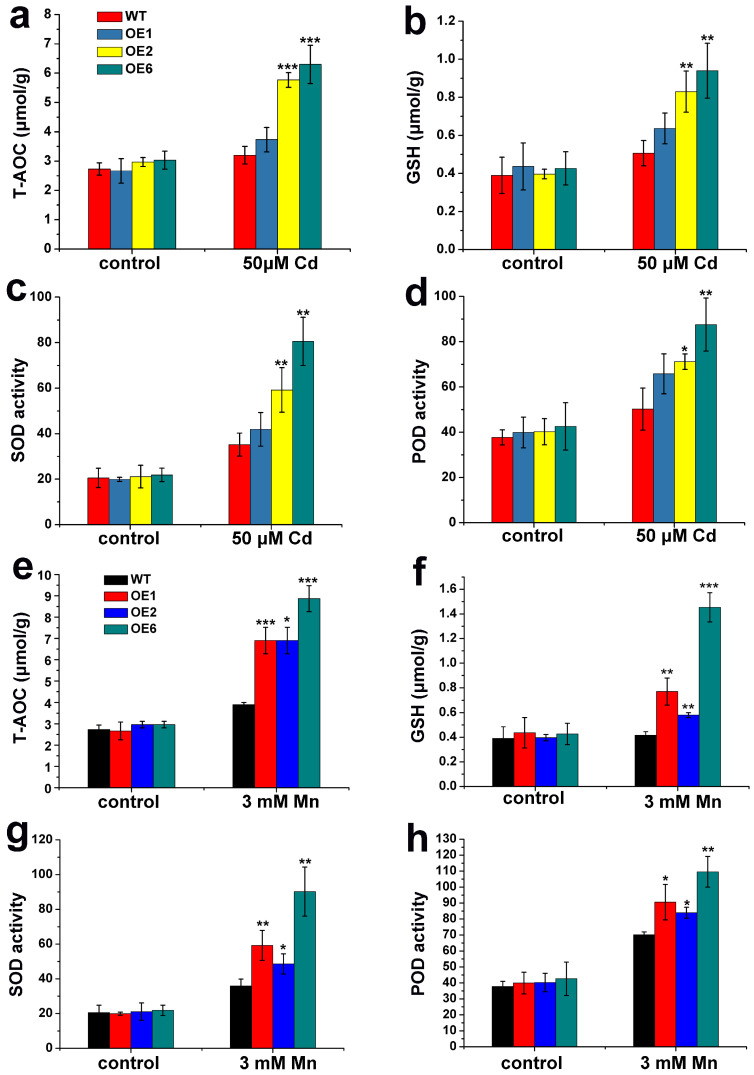
Physiological and biochemical indexes of *LcSHMT4* transgenic rice seedlings under heavy metal stress (50 μM CdCl_2_ or 3 mM MnSO_4_). (**a**–**d**) The data for Cd treatment. (**e**,**f**) The data for Mn treatment. (**a**,**e**) T-AOC, (**b**,**f**) GSH content, (**c**,**g**) SOD activity, and (**d**,**h**) POD activity. Values are mean ± standard error from three independent experiments. Asterisks indicate significant differences (* *p* < 0.05, ** *p* < 0.01, *** *p* < 0.001, *t*-test). The seedlings were cultivated on ½ MS solid medium for 7 days. The 7-day-old rice seedlings were treated with 50 μM CdCl_2_ and 3 mM MnSO_4_ in Hoaglands’ solution, and fresh seedlings (0.1 g) were collected for analyses of physiological and biochemical indexes.

The Cd and Mn tolerance of WT and *LcSHMT4* transgenic lines was determined by measuring a series of physiological and biochemical indexes. In the absence of Cd and Mn, there were no differences in the T-AOC value, GSH content, and SOD and POD activities between the WT and the transgenic rice lines (Figure 5). After the Cd treatment, the T-AOC values, GSH contents, and SOD and POD activities were significantly higher in OE2 and OE6 than in WT (*p* < 0.01). Although these indexes were also slightly higher in OE1 than in WT, the differences were not significant (Figure 5a–d). After the Mn stress treatment, the T-AOC values, GSH contents, and SOD and POD activities were significantly higher in OE1, OE2, and OE6 than in WT (Figure 5e–h, *p* < 0.05, *p* < 0.01, *p* < 0.001). These results indicate that Cd and Mn enhance SOD and POD activities in rice, and LcSHMT4 increases SOD and POD activities in transgenic plants while promoting GSH synthesis, thereby improving the antioxidant capacity.

**Figure 6:**
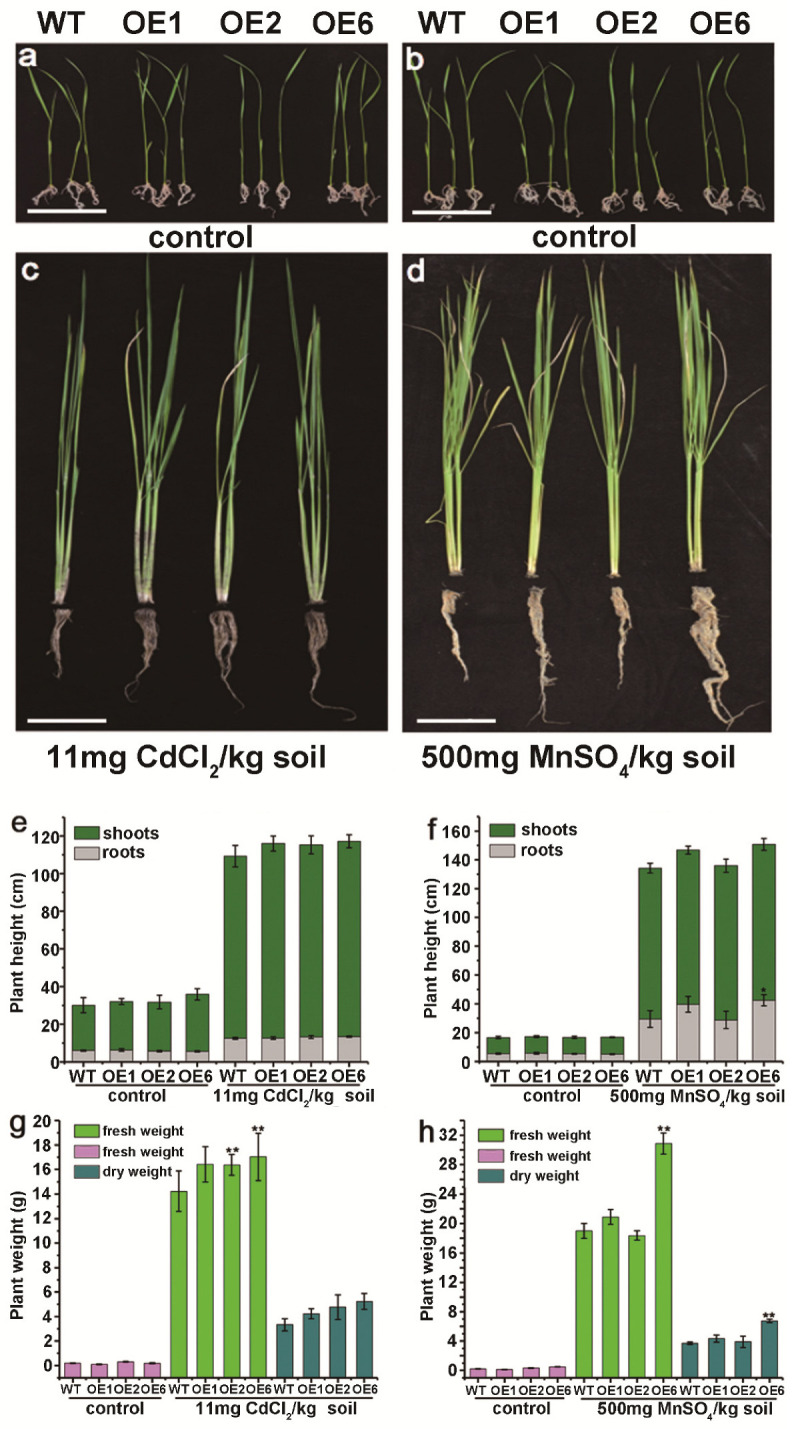
Plant growth of wild-type and *LcSHMT4*-expressing transgenic rice under Cd and Mn treatments. Seven-day-old seedlings with similar growth were planted in agricultural soil (mixture of loam soil and peat soil, quality ratio = 1:1) and then grown for 60 days. The 60-day-old seedlings were grown in 11 mg CdCl_2_/kg soil or 500 mg MnSO_4_/kg soil for 60 days under a 16 h light (28 °C)/8 h dark (26 °C) photoperiod with 70–80% humidity. (**a**–**d**) Phenotype; (**e**,**f**) plant height, (**g**,**h**) plant weight (Purple column: fresh weight of 7-day-old seedlings. Light green column: fresh weight of 67-day-old plants. Dark green column: dry weight of the 67-day-old plants). Scale bar: 10 cm. Values are mean ± standard error from three independent replicates. Asterisks indicate significant differences (** *p* < 0.01).

Tissue-cultured seedlings of WT and transgenic rice lines with similar heights were selected for culture in soil (Figure 6a,b). After Cd treatment, the root length was similar in the WT and transgenic seedlings, but shoot height was slightly lower in WT seedlings than in the transgenic lines (Figure 6c,e). The fresh weight of transgenic lines was higher than that of WT. The fresh weights of OE2 and OE6 were significantly higher than that of WT (*p* < 0.01), and the dry weight of the transgenic lines also was higher than that of WT during the vegetative growth stage (Figure 6g).

In the Mn treatment, the shoot height was almost the same in the WT and transgenic lines, but the root lengths of OE6 were significantly greater than that of WT, with the root length of OE6 being approximately 1.5-fold that of WT (Figure 6f, *p* < 0.05). The fresh and dry weights of OE1 and OE6 were higher than those of WT. These differences were significant between OE6 and WT, with the fresh and dry weight of OE6 being about 1.7-fold and 1.6-fold those of WT (Figure 6h, *p* < 0.05).

**Figure 7:**
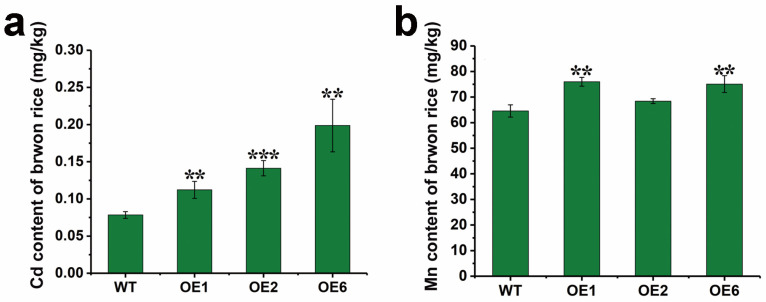
Cd or Mn contents in rice grains of control and transgenic lines. (**a**) Cd content in brown rice of plants subjected to 11 mg CdCl_2_/kg soil treatment; (**b**) Mn content in brown rice of plants subjected to 500 mg MnSO_4_/kg soil treatment. Values are mean ± standard error from three independent experiments. Asterisks indicate significant differences (** *p* < 0.01, *** *p* < 0.001, *t*-test). Seven-day-old seedlings were grown in agricultural soil with 11 mg CdCl_2_/kg soil and 500 mg MnSO_4_/kg soil for 120 days. Mature brown rice grains (0.5 g) were collected and the Cd or Mn contents were measured by ICP-MS.

In plants cultivated under the same Cd and Mn concentrations, the Cd or Mn contents in grains of mature plants were higher in the transgenic lines than in WT (Figure 7a,b, *p* < 0.01, *p* < 0.001), indicating that the grains from transgenic lines effectively accumulated these heavy metals.

### 2.6. LcSHM4 Regulates the Expression of Genes Encoding Cd and Mn Transporters

**Figure 8:**
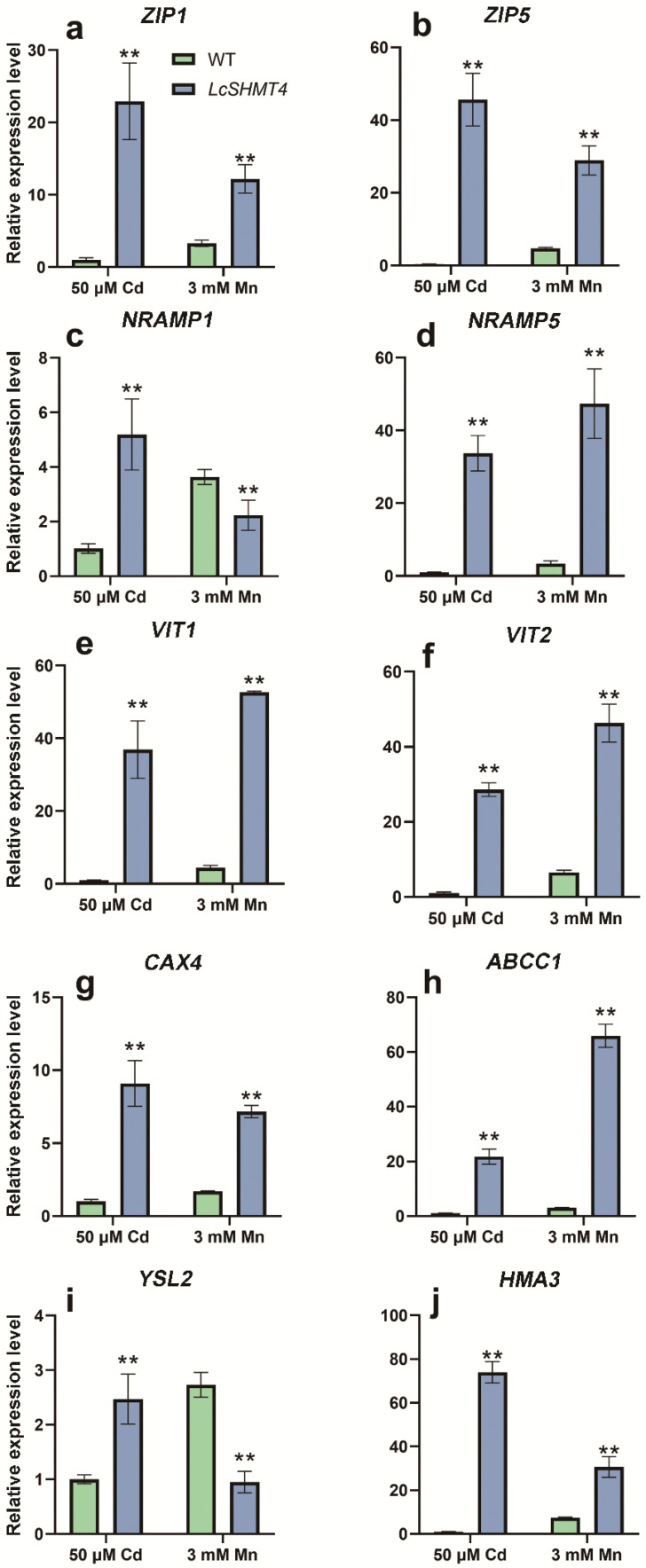
Transcript levels of 10 genes encoding Cd or Mn transporters in WT and transgenic *LcSHMT4* (OE2) seedlings under Cd or Mn treatments. The seeds of three transgenic lines (OE2) were germinated on ½ MS solid medium for 2 days in darkness at 37 °C, and then the seedlings were cultivated at 25 °C for 7 days with a 16 h light/8 h dark photoperiod (control group). The 7-day-old rice seedlings were treated with 50 μM CdCl_2_ and 3 mM MnSO_4_ in Hoaglands’ solution Total RNA was isolated from the WT and *LcSHMT4*-OE2 rice seedlings (0.1 g), and then relative gene transcript levels were determined by RT-qPCR. Values are means ± standard error of three independent samples. Asterisks indicate significant differences (** *p* < 0.01, *t*-test).

The transcript levels of 10 transporter-encoding genes (*OsZIP1*, *OsZIP5*, *OsNRAMP1*, *OsNRAMP5*, *OsVIT1*, *OsVIT2*, *OsCAX4*, *OsABCC1*, *OsYSL2*, and *OsHMA3*) were determined in WT and *LcSHMT4*-overexpressing transgenic rice (OE2) after 24 h of 50 μM CdCl_2_ or 3 mM MnSO_4_ treatments. The transcript levels of all these genes were significantly higher in OE2 plants than in WT plants after the Cd treatment (Figure 8; *p* < 0.01). After the Mn treatment, the transcript levels of *OsZIP1*, *OsZIP5*, *OsNRAMP5*, *OsVIT1*, *OsVIT2*, *OsCAX4*, *OsABCC1*, and *OsHMA3* were higher in OE2 than in WT (Figure 8a,b,d–h,g; *p* < 0.01), but those of *OsNRAMP1* and *OsYSL2* were significantly lower in OE2 than in WT (Figure 8c,i; *p* < 0.01). These results suggested that LcSHMT4, localized at the mitochondria, promotes the expression of genes encoding plasma membrane or tonoplast transporters in response to Cd or Mn stress.

## 3. Discussion

Previous studies have shown that sheepgrass can grow normally in saline-alkali soils with a pH of 8.5–11.5. Additionally, this grass exhibits adaptability to drought and low temperatures, demonstrating strong stress tolerance. These observations suggest that sheepgrass harbors high-quality stress-tolerance genes. However, genetic research on this species is limited, and few studies have explored the identity and mechanisms of its stress-tolerance genes [26]. The full genome sequence of sheepgrass was reported more than a decade ago [27]. The availability of this genomic data will facilitate research to functionally validate genes involved in stress tolerance and to use these genes in breeding programs to generate stress-resistant plants.

Several studies have functionally characterized genes related to stress tolerance from sheepgrass. Overexpression of the sheepgrass *LcCBF6* (C-repeat binding factor 6) gene in *Arabidopsis* significantly enhanced salt tolerance, indicative of its crucial role in improving tolerance to salinity [28]. In another study, transgenic *Arabidopsis* expressing sheepgrass *LcMYB1* exhibited higher proline levels compared with WT plants, suggesting that *LcMYB1* may regulate downstream genes involved in saline-alkali tolerance [29]. The drought-tolerance gene *LcP5CS1* from sheepgrass was confirmed to be involved in proline synthesis [30]. Additionally, transgenic *Arabidopsis* and rice expressing the sheepgrass *LcFIN2* gene showed improved cold stress tolerance [31].

Most of those studies focused on genes from sheepgrass related to drought, salt, and cold tolerance [28,29,30,31]. However, this grass also exhibits heavy metal tolerance, making it a useful resource for the discovery of genes related to this trait. Sheepgrass *LcNRAMP2* is expressed in the roots and encodes a transporter protein that participates in Cd and Mn ion transport. When transgenic rice plants expressing *LcNRAMP2* were subjected to heavy metal stress, LcNRAMP2 enhanced Cd and Mn tolerance and accumulation by sequestering excess Cd and Mn ions in the vacuole [24].

Previous studies have explored the functions of various SHMTs. Rice overexpressing *OsSHMT3* showed enhanced photosynthetic efficiency, increased accumulation of the osmo-regulatory substances glycine and serine, and stronger salt stress tolerance [32]. Cucumber (*Cucumis sativus*) overexpressing *CsSHMT3* showed increased photosynthetic efficiency, antioxidant enzyme activity, and proline content. Cucumber plants with silenced *SHMT3* accumulated higher levels of H_2_O_2_ and O_2_^−^ under drought stress, compared with WT, indicative of a greater ROS response and cellular damage [33]. Cotton (*Gossypium herbaceum*) *GhSHMT11* is localized at the mitochondria. Silencing of *GhSHMT11* using the virus-induced gene silencing system led to increased ROS accumulation, a reduced photosynthetic rate in the leaves, and a notable decrease in biomass. Heterologous overexpression of *GhSHMT11* in *Arabidopsis* improved its salt tolerance by reducing ROS accumulation [25]. Alfalfa (*Medicago sativa*) *MsSHMT* family genes were found to be induced by salt and drought stresses [34]. In our study, *LcSHMT4* was first demonstrated to exhibit upregulated expression levels by Cd and Mn treatments and increased the Cd and Mn tolerance of transgenic yeast and rice (Figure 1, Figure 2, Figure 3, Figure 4, Figure 5 and Figure 6).

When plants are subjected to adverse environmental stresses, excessive ROS accumulate in cellular organelles such as mitochondria and cause damage by disrupting photorespiration and physiological metabolism. Plants use two main mechanisms to eliminate ROS, the enzymatic pathway and the non-enzymatic pathway. In the enzymatic pathway, SOD, POD, and catalase (CAT) form the first line of defense. Superoxide dismutase converts highly reactive superoxide radicals (O_2_^−^) into H_2_O_2_, which is then rapidly broken down into water (H_2_O) by POD and CAT, minimizing toxicity to plant cells [35]. Glutathione is another critical component of the plant antioxidant system [36]. Its reduced form, GSH, functions both as a direct ROS scavenger in enzymatic reactions and, more importantly, as a key point in the non-enzymatic ascorbate–glutathione (AsA-GSH) cycle, which regulates intracellular redox balance. The AsA-GSH cycle, the primary ROS-scavenging mechanism in plants, predominantly functions in chloroplasts, but its activity has also been detected in the cytoplasm and mitochondria. In this cycle, H_2_O_2_ is reduced in a reaction involving AsA and GSH, which functions as the redox intermediate. The non-enzymatic pathway can provide protection against heavy-metal-induced oxidative damage. For instance, under Cd stress, the AsA-GSH cycle was found to alleviate oxidative damage by the addition of sulfur-containing compounds, which increased phytochelatins (PCs) and PCs–Cd complexes, and reduced Cd accumulation in lettuce [37]. Hydrogen sulfide significantly alleviated the oxidative damage caused by Mn stress on apple seedlings, mainly by enhancing the activity of antioxidant enzymes and upregulating the ASA-GSH cycle [38]. In our study, compared with control rice plants, those overexpressing *LcSHMT4* exhibited higher height and weight, increased T-AOC and GSH content, and higher activities of SOD and POD under Cd and Mn stress (Figure 5 and Figure 6). This suggests that LcSHMT4 enhances heavy metal tolerance through improving antioxidant enzyme activity, thereby mitigating ROS-induced toxicity.

In some species, SHMTs function in combination with other proteins and/or influence the expression of other genes that participate in stress responses. For instance, co-overexpression of *SHMT* and formate dehydrogenase in tobacco enhanced formaldehyde (HCHO) metabolism and absorption of liquid HCHO, thereby promoting plant growth [39]. In another study, the Cd tolerance mechanism of a Cd-tolerant rice mutant, *cadt1*, was investigated in detail. The mutant exhibited higher transcript levels of the sulfate transporter gene *OsSULTR1* and the sulfur-deficiency-induced gene *OsSDI1*, leading to increased accumulation of sulfur and selenium in rice grains. Additionally, *cadt1* showed elevated levels of GSH and phytochelatins—key compounds for ROS scavenging—resulting in enhanced Cd tolerance. These findings suggested that CADT1 negatively regulates the expression of *OsSULTR1* and *OsSDI1*, and so its mutation could increase Cd tolerance [40]. Our results suggest that LcSHMT4 may regulate the expression of genes encoding Cd–Mn influx transporters (Figure 8), leading to enhanced uptake and segregation of Cd and Mn within the cell. This would maintain the Cd and Mn balance in the cytoplasm and ultimately lead to increased transport of these metal ions from the roots to the shoots so that more Cd and Mn are stored in the seeds.

**Figure 9:**
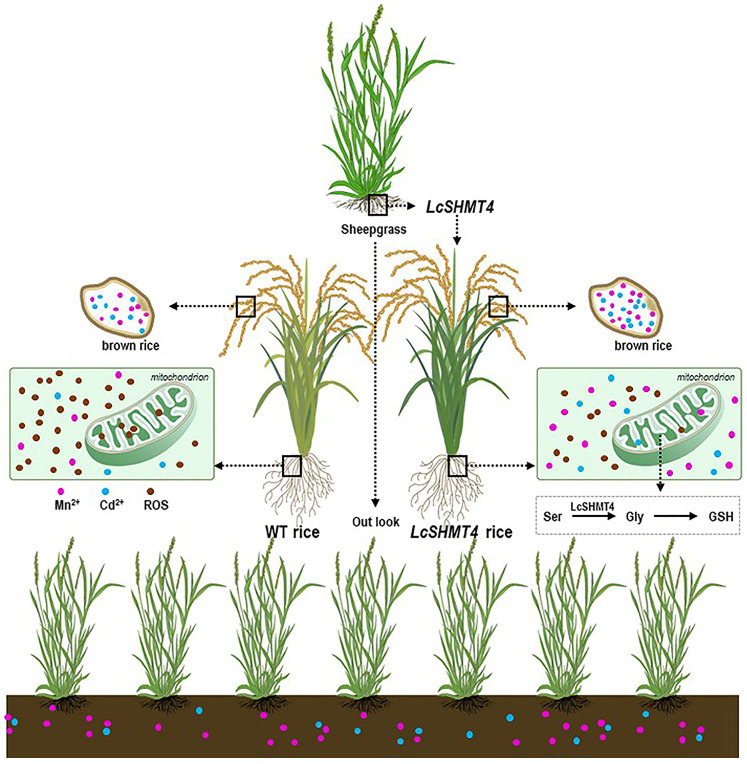
Proposed role of *LcSHMT4* in Cd and Mn tolerance and accumulation. *LcSHMT4* may be involved with catalyzing glycine production in mitochondria. Glycine as a precursor is used for synthesis of GSH, which scavenges intracellular ROS. The increase in SOD and POD activities further strengthen the antioxidant defense system, collectively improving heavy metal tolerance. *LcSHMT4* may influence the transport of Cd and Mn and ultimately leads to their accumulation in the fruits through interaction with transporters.

We found that *LcSHMT4* transcript levels increased in sheepgrass under Cd and Mn stress and that its heterologous expression in both rice and yeast enhanced their Cd and Mn tolerance (Figure 1, Figure 2 and Figure 5, and 6). In addition, rice plants overexpressing *LcSHMT4* showed increased tolerance to Cd and Mn and increased Cd and Mn accumulation in the brown rice grains (except OE2, Figure 5, Figure 6 and Figure 7). On the basis of these results and those of previous studies on SHMTs, we propose a model for how LcSHMT4 improves the heavy metal tolerance of yeast and rice. The underlying mechanism may involve LcSHMT4 catalyzing glycine production in mitochondria. Glycine then serves as a precursor for the synthesis of GSH, which scavenges intracellular ROS. At the same time, elevated SOD and POD activities further strengthen the antioxidant defense system, collectively improving heavy metal tolerance. In addition, LcSHMT4 may influence the transport of Cd and Mn, and ultimately leads to their accumulation in the fruits through interaction with transporters (Figure 9).

## 4. Materials and Methods

### 4.1. Cultivation of Sheepgrass Seedlings

Sheepgrass seeds were obtained from the Institute of Forage and Grassland Sciences from Heilongjiang Academy of Agricultural Sciences (Harbin, Heilongjiang, China). The seeds were sterilized and cultured on ½ Murashige and Skoog (MS) solid medium (3% *w*/*v* sucrose, 0.2% *w*/*v* phytagel, pH 5.8). The sterilized seeds were vernalized at 4 °C for 7 days then cultivated at 25 °C and 40–60% humidity under a 12 h/12 h (light/dark) photoperiod with 150 µE m^−2^ s^−1^ light intensity.

### 4.2. Isolation of LcSHMT4 and Bioinformatics Analysis

Total RNA was isolated from 5-day-old sheepgrass seedlings. The seedlings were ground in liquid nitrogen with a mortar and pestle, and the total RNA was extracted using a TransZol^TM^ Up Plus RNA Kit (TransGen Biotech, Beijing, China). Then, cDNA was synthesized using the Hifair^®^ III 1st Strand cDNA Synthesis Kit (gDNA digester plus) (Yeasen Biotechnology, Co., Ltd., Shanghai, China). The *LcSHMT4* gene was amplified with specific primers (LcSHMT4-F and LcSHMT4-R). The sequence of *LcSHMT4* was used to search for sequences with high homology from other species using the BLASTp function at the NCBI website (https://www.ncbi.nlm.nih.gov/). Then, sequence alignment of all SHMT4s was performed using ClustalX 1.81 and GeneDoc 2.7 software. The MEME website (http://meme-suite.org/) was used to predict and analyze the conserved motifs of LcSHMT4 and other protein sequences (setting the number of motifs to 5). A phylogenetic analysis was conducted with MEGA5.0 software, using the LcSHMT4 sequence from sheepgrass and other related sequences.

### 4.3. Yeast Transformation and Heavy Metal Tolerance and Accumulation in Transformed Strains

The pYES2-LcSHMT4 recombinant plasmid was constructed using the ClonExpress Ultra One Step Cloning Kit V3 (Vazyme Biotech Co., Ltd., Nanjing, China). The *LcSHMT4* gene was amplified using specific primers (LcSHMT4-pYES2-F and LcSHMT4-pYES2-R) and inserted into the pYES2 vector via the *Eco*RI and *Bam*HI restriction sites. The empty vector pYES2 (control) and pYES2-LcSHMT4 were transformed into the wild-type yeast strain BY4741 and mutant yeast strains YK44 (sensitive to Cd) [41] and *smfI* (sensitive to Mn) [42] using the PEG/LiAC method. The pYES2 and *LcSHMT4* transgenic BY4741 cells were adjusted to OD_600_ = 0.5 (10^0^) and then diluted to 10^−1^, 10^−2^, and 10^−3^ before spreading 4 μL of each dilution onto YPD or yeast extract/peptone/galactose (YPG) solid medium containing different concentrations of CuSO_4_, Pb(NO_3_)_2_, CdCl_2_, ZnSO_4_, CoCl_2_, NiSO_4_, MnSO_4_, and FeSO_4_. The pYES2 and *LcSHMT4* transgenic YK44 and *smfI* yeast strains were cultured in YPD or YPG media with CdCl_2_ and MnSO_4_, respectively. The growth of yeast cells was observed and photographed at 30 °C for 2–7 days.

The mutant yeast cells were suspended in YPG medium to an OD_600_ = 0.1. All cells were grown in media containing 0 or 50 μM CdCl_2_ and 0 or 3 mM MnSO_4_. The cell growth curves were constructed using measurements of OD_600_ recorded every 4 h from 0 to 48 h. The OD_600_ value was measured using a SpectraMax Absorbance Reader (Molecular Devices, Sunnyvale, CA, USA). Equal optical density (OD_600_ = 0.5) of pYES2-LcSHMT4 and pYES2-transformed YK44 and *smfI* cells were treated with 50 μM CdCl_2_ or 3 mM MnSO_4_ for 2 days. The test cells were washed, dried, and digested, and then their Cd and Mn contents were measured by inductively coupled plasma–mass spectrometry (ICP-MS) at the Nanjing Convinced-test Technology Co., Ltd. (Wuxi, China).

### 4.4. Expression Analysis of LcSHMT4

For the heavy metal treatments, 5-day-old sheepgrass seedlings (0.1 g) were treated with 50 μM CdCl_2_ and 3 mM MnSO_4_, and then shoot and root samples were collected at 0 h, 6 h, 12 h, 24 h, and 48 h. All samples were immediately frozen in liquid nitrogen and ground into a powder for subsequent gene expression analyses. After extraction of total RNA from the samples, cDNA was synthesized using the Hifair^®^ III 1st Strand cDNA Synthesis SuperMix for qPCR kit (gDNA digester plus). The transcript level of *LcSHMT4* was determined by RT-qPCR with gene-specific primers, using Hieff qPCR SYBR Green Master Mix (Yeasen Biotechnology) and Actin as the internal control. The relative gene transcript levels were calculated using the 2^−ΔΔCT^ method as previously described [26].

### 4.5. Localization of LcSHMT4

The *LcSHMT4* gene was amplified with LcSHMT4-specific primers (LcSHMT4-1300-F and LcSHMT4-1300-R) and inserted into pCAMBIA1300-GFP using the ClonExpress Ultra One Step Cloning Kit V3 (Vazyme) via the *Bam*HI and *Sal*I restriction sites. The recombinant plasmid was transferred into *Agrobacterium* tumefaciens GV3101, which was then injected into 10 leaves of tobacco. The tobacco was first cultured in the dark for 24 h and then cultivated under light for 48 h to transiently express LcSHMT4. Leaves infected with *Agrobacterium* were stained with a 0.5 μM MitoTracker Red CMXRos, a mitochondria-specific dye, for 40 min [25,43]. The green GFP and dye signals in the leaf epidermis cells were observed and photographed under a confocal laser scanning microscope (TCS-SP8, Leica, Wetzlar, Germany).

### 4.6. Plant Transformation and Heavy Metal Tolerance and Content Analyses

The pCAMBIA1300-LcSHMT4 recombinant plasmid was transformed into wild-type (WT) rice plants (*Oryza sativa* L. ssp. japonica cv. Nipponbare) by Wimi Biotechnology Co., Ltd. (Wuxi, China). The presence of the plasmid in the transgenic rice seedlings was confirmed by semi-PCR and RT-qPCR. The seeds of three transgenic lines (OE1, OE2, and OE-6) were germinated on ½ MS solid medium for 2 days in darkness at 37 °C, and then the seedlings were cultivated at 25 °C for 7 days with a 16 h light/8 h dark photoperiod (control group). The 7-day-old rice seedlings (0.1 g) were treated with 50 μM CdCl_2_ and 3 mM MnSO_4_ in Hoaglands’ solution (pH5.8) [44], and fresh samples of the seedlings, including shoot and roots (0.1 g), were collected for analyses of physiological and biochemical indexes, including the total antioxidant capacity (T-AOC), the content of reduced glutathione (GSH), and activities of superoxide dismutase (SOD) and peroxidase (POD). These analyses were conducted using kits from Solarbio (Beijing, China).

In addition, 7-day-old seedlings with similar growth were planted in agricultural soil (mixture of loam soil and peat soil, quality ratio = 1:1) and then grown for 60 days. The seedlings at the vegetative growth stage were grown in heavy-metal-contaminated soil (11 mg CdCl_2_/kg soil and 500 mg MnSO_4_/kg soil) for 60 days under a 16 h light (28 °C)/8 h dark (26 °C) photoperiod with 70–80% humidity. The phenotypes and weight of the WT and transgenic rice lines were compared at the vegetative growth stage. In addition, after 120 days, mature brown rice grains were collected from the WT and transgenic rice plants. Mature brown rice grains (0.5 g) were added to 5 mL of nitric acid and soaked overnight. Then, all samples were kept at 80 °C for 2 h, 120 °C for 2 h, and, finally, the temperature was raised to 160 °C for 4 h. After cooling to room temperature, the digestion solution was adjusted to a volume of 25 mL. The Cd and Mn contents were measured by ICP-MS at the Nanjing Convinced-test Technology Co., Ltd.

### 4.7. Expression of Genes Encoding Cd–Mn Transporters in Rice

To explore the regulatory effect of LcSHMT4 on other proteins involved in Cd and Mn tolerance and transport, the sequences of rice genes encoding transporters localized to the plasma membrane (*OsZIP1*, *OsZIP5*, *OsNRAMP1*, *OsNRAMP5*, and *OsYSL2*) and vacuole membrane (*OsVIT1*, *OsVIT2*, *OsCAX4*, *OsABCC1*, and *OsHMA3*) were obtained by searching the GenBank database. The WT and transgenic rice (OE2, 0.1 g) seedlings were treated with Hoagland’s solution containing 50 μM CdCl_2_ and 3 mM MnSO_4_. Total RNA was isolated from the WT and *LcSHMT4*-OE2 rice plants, and then relative gene transcript levels were determined by RT-qPCR.

### 4.8. Statistical Analysis

The reported values are mean ± standard error from three dependent biological replicates. The *t*-test was used to analyze the significance of differences (* *p* < 0.05, ** *p* < 0.01, *** *p* < 0.001). Figures were generated using Origin 8 and Photoshop CS2.

## 5. Conclusions

In summary, we analyzed the growth, physiology, and mechanism of transgenic yeast and rice expressing *LcSHMT4* to explore its role in Cd and Mn detoxification and tolerance. Therefore, we identified genetic resources that will be useful for breeding heavy-metal-accumulating and heavy-metal-tolerant plants. Our results provide theoretical support for mining stress-tolerance genes and for developing phytoremediation strategies to detoxify heavy-metal-contaminated soils.

## Data Availability

The original contributions presented in this study are included in the article. Further inquiries can be directed to the corresponding author.

## Supplementary Materials

The following supporting information can be downloaded at: https://www.mdpi.com/article/10.3390/plants15010091/s1. Table S1: List of all primer sequences used in this study; Figure S1 (See Refs. [45,46,47,48,49,50,51,52,53,54]): Sequence alignment of serine hydroxymethyltransferase 4 (*LcSHMT4*) from sheepgrass and SHMT4s of other species; Figure S2: The neighbor-joining phylogenetic relationships among *LcSHMT4* from sheepgrass and SHMT4s from other species; Figure S3: Expression and identification of *LcSHMT4* gene in transgenic rice.
