# Supplementary material for: LcSHMT4 from Sheepgrass Improves Tolerance to Cadmium and Manganese and Enhances Cd and Mn Accumulation in Grains
Source: Plants (Basel). 2025 Dec 27;15(1):91. doi: 10.3390/plants15010091 (PMC12787419; doi:10.3390/plants15010091)
Supplement: Supplementary file 1 [file plants-15-00091-s001.zip › plants-4027191-supplementary.pdf]

Article

LcSHMT4 from sheepgrass improves tolerance to cadmium and manganese and enhances Cd and Mn accumulation in grains

Jianli Wang [1,+], Guili Di [3,+], Yuanyuan Lin [2], Linlin Mu [1], Xu Zhuang [1], Dongmei Zhang [1], Weibo Han [1], Tuanyao Chai [4], Aimin Zhou [2,*], Kun Qiao [2,*]

[1] Institute of Forage and Grassland Sciences, Heilongjiang Academy of Agricultural Sciences, Harbin, 150086, China jianli@haas.cn (J.L.W.); mulinys@163.com (L.L.M.); 13030086918@163.com (X.Z.); zhd_mei@163.com (D.M.Z.); alclever@163.com (W.B.H.)

[2] College of Horticulture and Landscape Architecture, Northeast Agricultural University, Harbin, 150030, China linyuanyuan2020@126.com (Y.Y.L.); aiminzhou@neau.edu.cn (A.M.Z.); kunqiao@neau.edu.cn (K.Q.)

[3] Industrial Crops Institute, Heilongjiang Academy of Agricultural Sciences, Harbin, 150086, China diguili59@163.com (G.L.D.)

[4] College of Life Science, University of the Chinese Academy of Sciences, Beijing, 100049, China tychai@ucas.ac.cn (T.Y.C.)

[+] Jianli Wang and Guili Di contributed equally to this work.

* Correspondence: aiminzhou@neau.edu.cn; kunqiao@neau.edu.cn

This supporting information contains three figures and one table.
Figures: Figure S1-S3
Tables: Table S1

Added Materials and Methods

In yeast and plant tolerance assay, high-concentration metal concentrate solution was prepared, and then filtered it for sterilization. After the culture medium is sterilized, the metal concentrate solution was added into the culture medium to reach the appropriate concentration according to the total volume of the culture medium.

The plasma membrane and vacuole membrane (*OsZIP1*, *OsZIP5*, *OsNRAMP1*, *OsNRAMP5*, *OsVIT1*, *OsVIT2*, *OsCAX4*, *OsABCC1*, *OsYSL2*, and *OsHMA3*) were obtained by searching the GenBank database. The accession numbers were *OsZIP1* (FJ940751.1), *OsZIP5* (AB126087.1), *OsNRAMP1* (AAB36424.1), *OsNRAMP5* (AB698459.1), *OsVIT1* (LC545390.1), *OsVIT2* (CT828611.1), *OsCAX4* (AB859021.1), *OsABCC1* (XM_015779966.3), *OsYSL2* (NP_001403724.1), *OsHMA3* (XM_015791882.3), and *Osactin1* (AK071586.1).

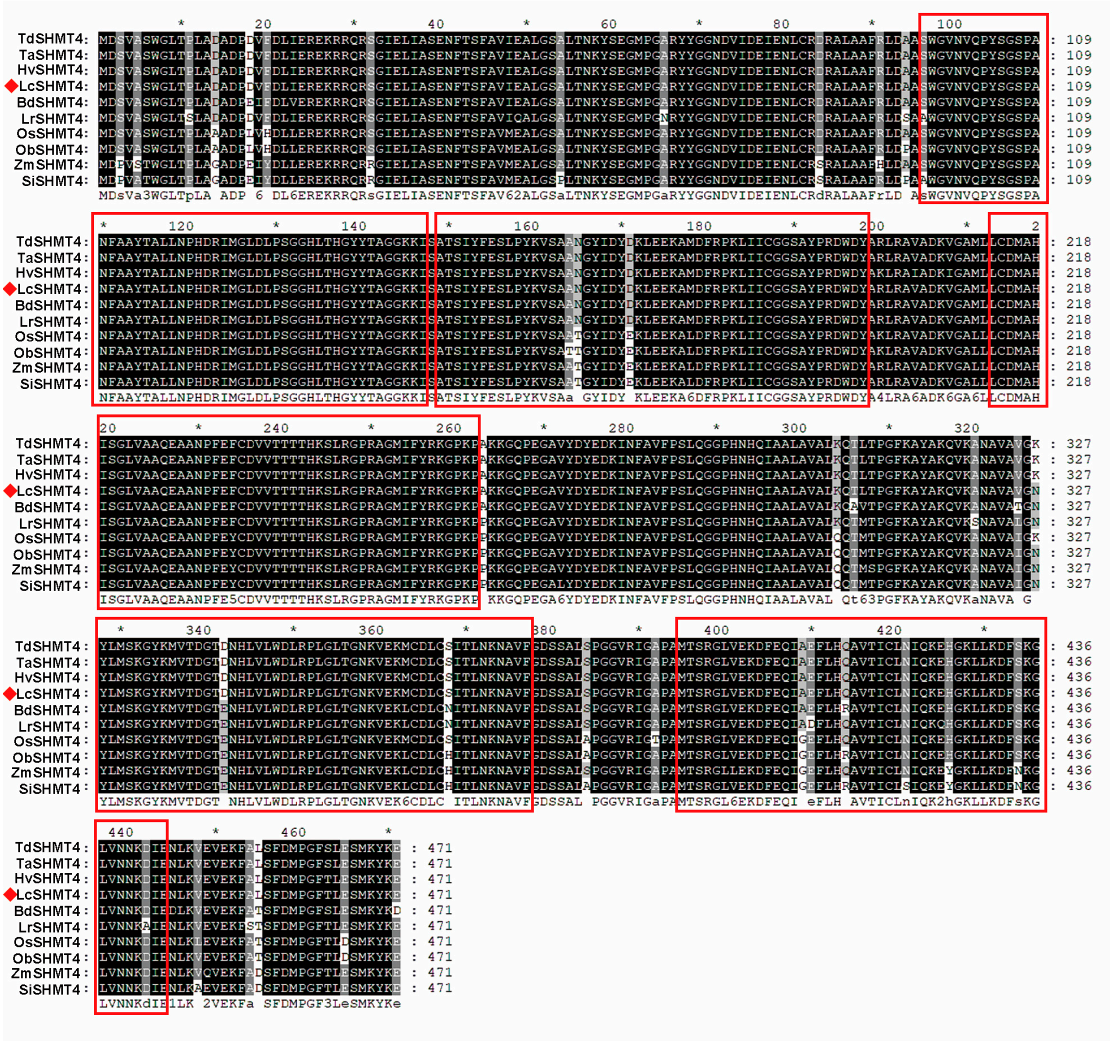

Figure S1 Sequence alignment of serine hydroxymethyltransferase 4 (LcSHMT4, red rhombus) from sheepgrass and SHMT4s of other species. The conserved domains are framed by red boxes. Lc, *Leymus chinensis* (PV719638.1); Td, *Triticum dicoccoides* (XM_037623682.1); Ta, *Triticum aestivum* (XM_044603316.1); Hv, *Hordeum vulgare* (AK362400.1); Bd, *Brachypodium distachyon* (XM_003577536.4); Lr, *Lolium rigidum* (XM_047234955.1); Os, *Oryza sativa* (XM_015761639.3); Ob, *Oryza brachyantha* (XM_006662863.3); Zm, *Zea mays* (EU960962.1); Si, *Setaria italica* (XM_004979181.4).

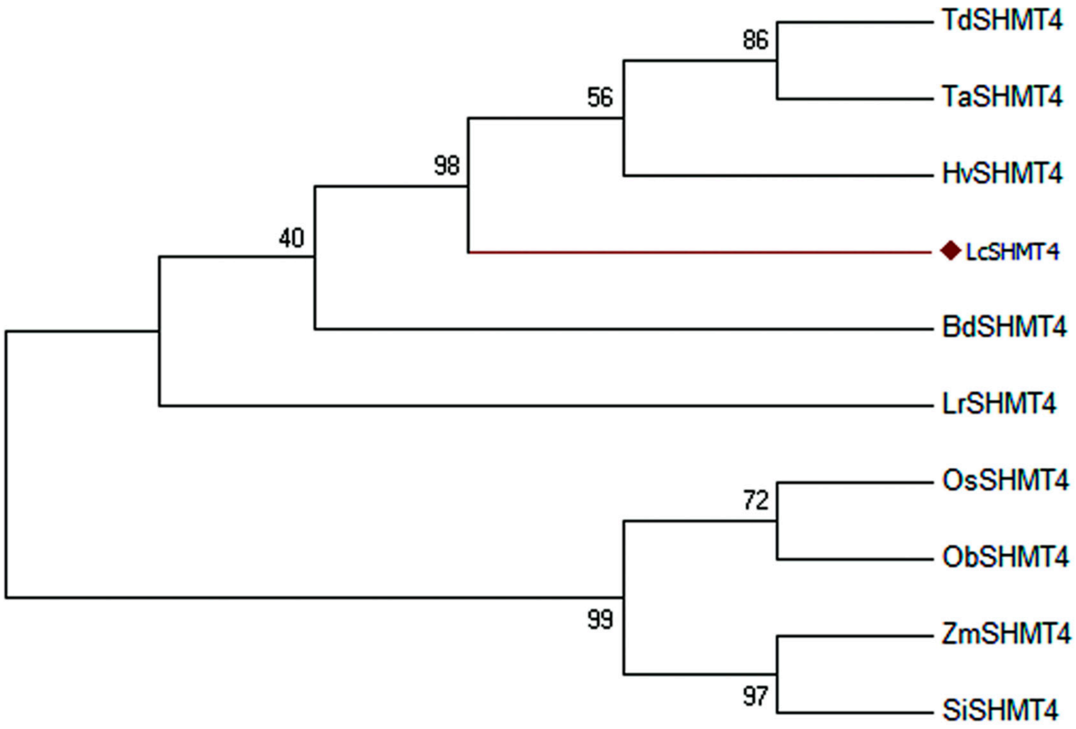

Figure S2 The neighbor-joining phylogenetic relationships among LcSHMT4 from sheepgrass and SHMT4s from other species. Bootstrap values were calculated 1,000 times; values < 50% are not shown. Lc, *Leymus chinensis* (PV719638.1); Td, *Triticum dicoccoides* (XM_037623682.1); Ta, *Triticum aestivum* (XM_044603316.1); Hv, *Hordeum vulgare* (AK362400.1); Bd, *Brachypodium distachyon (XM_003577536.4)*; Lr, *Lolium rigidum* (XM_047234955.1); Os, *Oryza sativa* (XM_015761639.3); Ob, *Oryza brachyantha* (XM_006662863.3); Zm, *Zea mays* (EU960962.1); Si, *Setaria italica* (XM_004979181.4).

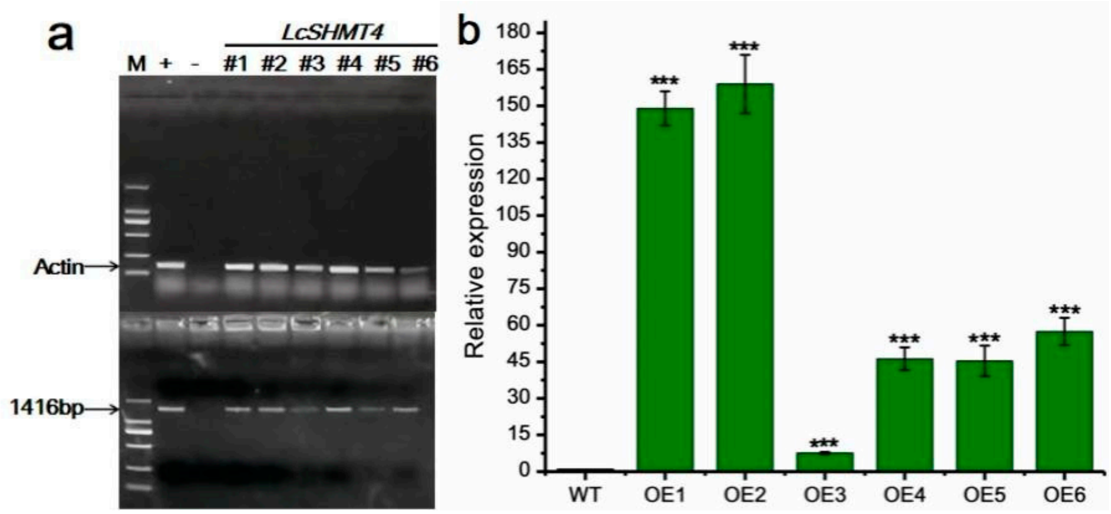

Figure S3 Expression and identification of *LcSHMT4* gene in transgenic rice. **a**: The transcript levels of six *LcSHMT4* transgenic rice (7-day-old seedlings) through semi-PCR analysis. **b**: The transcript levels of six *LcSHMT4* transgenic rice through real-time quantitative PCR analysis. WT, wild type rice; #1, OE1; #2, OE2; #3, OE3; #4, OE4; #5, OE5; #6, OE6. Twenty potential *LcSHMT4* transgenic rice were harvested, and the seeds of six transgenic lines (OE1, OE2, OE3, OE4, OE5, and OE-6) were germinated on ½ MS solid medium for 2 days in darkness at 37 °C, and then the seedlings were cultivated at 25 °C for 7 days with a 16-h light:8-h dark photoperiod. *LcSHMT4* transgenic rice; Significant differences were analyzed by *t*-test, and indicated using asterisks (***$p < 0.001$).

Table S1 List of primer sequences used in this study.

Primer Name	Sequence(5′-3′)
LcSHMT4-F	ATGGACTCGGTCGCGTCGTGGG
LcSHMT4-R	CTACTCCTTGTACTTCATGCTCTC
LcSHMT4-pYES2-F	GCCGCCAGTGTGCTGGAATTCATGGACTCGGTCGCG
LcSHMT4-pYES2-R	GCGGCCGTTACTAGTGGATCCCTACTCCTTGTACTTC
LcSHMT4-1300-F	GAGCTCGGTACCCGGGGATCCATGGACTCGGTCGCG
LcSHMT4-1300-R	GCCCTTGCTCACCATGTCGACCTCCTTGTACTTCATG
LcActin2-qF	TACGACCAGGAGATGGAGACC
LcActin2-qR	GAAGGAAGGCTGGAAGAGGAC
LcSHMT4-qF	GGTGCTCCCGCAATGACTTC

LcSHMT4-qR	CCGTGCTCCTTCTGGATGTT
OsActin5-qF	CTTGCTTTTGGCATTGACG
OsActin5-qR	AAGTTGCTCCATTAGTTCCTCC
OsABCC1-qF [52]	ATCGGTGGTCCTTCGTGGAAC
OsABCC1-qR	TGTTAGGTCACCACCTGGGAG
OsZIP1-qF [45]	AGTTCCTCGAGCGCTCCTGC
OsZIP1-qR	CAAGAACCCGAGGTCCAGTGC
OsHMA3-qF [53]	TCGCCGAGAACTCGACGGTG
OsHMA3-qR	GCTTCCACCATTGCTCAAGGC
OsZIP5-qF [46]	AGCCGGAGACGGACGTGTTC
OsZIP5-qR	TGTCGACGATGAGCGTGCCG
OsNRAMP5-qF [54]	ACAAGTGCGCCAACCTCAGC
OsNRAMP5-qR	TCAGGTTCCGAAGCCACTTCC
OsVIT1-qF [50]	ACGTCATCATGGGCGTCTCCG
OsVIT1-qR	CTGGTAATGGTCTGCCTCGCTC
OsCAX4-qF [51]	CTGAGATCTCCAAACGGGAAGC
OsCAX4-qR	CTGCTGAATTCCCCACCACTG
OsVIT2-qF [50]	AGCAGGAGGAGATCGACACC
OsVIT2-qR	TCCGGCTTCTCCAGTCCCAAC
OsNRAMP1-qF [47, 48]	TCGTCATCATCGGGATAAAC
OsNRAMP1-qR	TGTGCTTTCTCGGTGTCG
OsYSL2-qF [49]	TGCCTATGGAACTGGGCTAAC
OsYSL2-qR	AAAGTCAGTGGAGCGATTATGC
OsActin1-qF	GATCTTGCTGGCCGAGACCTC
OsActin1-qR	CAGAACCTCAGGGCACCTGAAC

References

45. Ramesh, S.A.; Shin, R.; Eide, D.J.; Schachtman, D.P. Differential metal selectivity and gene expression of two zinc transporters from rice. *Plant Physiol.* **2003**, *133*, 126–134.
46. Lee, S.; Jeong, H.J.; Kim, S.A.; Lee, J.; Guerinot, M.L.; An, G. OsZIP5 is a plasma membrane zinc transporter in rice. *Plant Mol. Biol.* **2010**, *73*, 507–517.
47. Takahashi, R.; Ishimaru, Y.; Senoura, T.; Shimo, H.; Ishikawa, S.; Arao, T.; Nakanishi, H.; Nishizawa, N.K. The OsNRAMP1 iron transporter is involved in Cd accumulation in rice. *J. Exp. Bot.* **2011**, *62*, 4843–4850.
48. Chang, J.D.; Huang, S.; Yamaji, N.; Zhang, W.W.; Ma, J.F.; Zhao, F.J. OsNRAMP1 transporter contributes to cadmium and manganese uptake in rice. *Plant Cell Environ.* **2020**, *43*, 2476–2491.
49. Ishimaru, Y.; Masuda, H.; Bashir, K.; Inoue, H.; Tsukamoto, T.; Takahashi, M.; Nakanishi, H.; Aoki, N.; Hirose, T.; Ohsugi, R.; Nishizawa, N.K. Rice metal-nicotianamine transporter, OsYSL2, is required for the long-distance transport of iron and manganese. *Plant J.* **2010**, *62*, 379–390.
50. Zhang, Y.; Xu, Y.H.; Yi, H.Y.; Gong, J.M. Vacuolar membrane transporters OsVIT1 and OsVIT2 modulate iron translocation between flag leaves and seeds in rice. *Plant J.* **2012**, *72*, 400–410.

51. Zou, W.L.; Chen, J.G.; Meng, L.J.; Chen, D.D.; He, H.H.; Ye, G.Y. The rice cation/H^+ exchanger family involved in Cd tolerance and transport. *Int. J. Mol. Sci.* **2021**, *22*, 8186.
52. Song, W.Y.; Yamaki, T.; Yamaji, N.; Ko, D.; Jung, K.H.; Fujii-Kashino, M.; An, G.; Martinoia, E.; Lee, Y.; Ma, J.F. A rice ABC transporter, OsABCC1, reduces arsenic accumulation in the grain. *Proc. Natl. Acad. Sci. USA* **2014**, *111*, 15699–15704.
53. Miyadate, H.; Adachi, S.; Hiraizumi, A.; Tezuka, K.; Nakazawa, N.; Kawamoto, T.; Katou, K.; Kodama, I.; Sakurai, K.; Takahashi, H.; Satoh-Nagasawa, N.; Watanabe, A.; Fujimura, T.; Akagi, H. OsHMA3, a P_{1B}-type of ATPase affects root-to-shoot cadmium translocation in rice by mediating efflux into vacuoles. *New Phytol.* **2011**, *189*, 190–199.
54. Ishimaru, Y.; Bashir, K.; Nakanishi, H.; Nishizawa, N.K. OsNRAMP5, a major player for constitutive iron and manganese uptake in rice. *Plant Signal. Behav.* **2012**, *7*, 763–766.
